# Hepatitis B co-infection in HIV-infected patients receiving antiretroviral therapy at the TC Newman Anti Retroviral Treatment Clinic in Paarl, Western Cape

**DOI:** 10.4102/sajhivmed.v17i1.336

**Published:** 2016-05-20

**Authors:** Jeanmari King, Dirk T. Hagemeister

**Affiliations:** 1 Division of Family Medicine and Primary Care, Stellenbosch University, South Africa; 2 District Health Services, Western Cape Department of Health, South Africa; 3 Department of Family Medicine, University of the Free State, South Africa; 4 Universitas Academic Hospital, Bloemfontein, South Africa

## Abstract

**Background:**

Hepatitis B virus (HBV) and human immunodeficiency virus (HIV) co-infection in South Africa is estimated to be between 5% and 23%; however, only limited evidence is available. Co-infection increases the risk of chronification of HBV, liver cirrhosis and death.

**Objective:**

To assess the HBV and/or HIV co-infection rate amongst the adult antiretroviral treatment cohort at the TC Newman ART Clinic in Paarl, Western Cape.

**Methods:**

In a retrospective, cross-sectional study, the routine hepatitis B surface antigen screening results for all adult HIV patients who were started on antiretroviral treatment over a period of 19 months were collected and analysed for gender, CD4 count and age.

**Results:**

Amongst the 498 participants (60% female participants), the Hepatitis B surface Antigen positivity rate was 7.6%. Male gender, age between 50 and 59 years and a low CD4 count were correlated with higher rates.

**Conclusion:**

Useful insight could be obtained by analysing routine data. The prevalence of almost 8% confirms the need for testing of HIV-positive patients for hepatitis B.

## Introduction

### Hepatitis B-HIV co-infection in global perspective

Hepatitis B virus (HBV), the most serious type of viral hepatitis, remains a public health problem worldwide, with an estimated 400 million cases of chronic infections and possibly as many as 6 million people co-infected with the human immunodeficiency virus (HIV) and HBV.^1^ HBV-HIV co-infections commonly occur because of their endemicity in the same world regions and shared routes of transmission.^2,3^ The rates for HBV co-infection in HIV-positive people are given as 5% – 30%, depending on geographic regions, and the co-infection is associated with an increased risk of liver cirrhosis, end-stage liver disease, and death, with low CD4 counts or concomitant alcohol use being additional risk factors.^4^ For two large European and North American HIV treatment cohorts, the prevalence of HBV co-infection has been given as 8.7%^4^ and 7.6%,^5^ respectively. Studies in South Africa have reported a HBsAg positivity rate of 19.8% in a mining/industrial antiretroviral treatment (ART) cohort,^6^ 4.8% and 6.5% in two urban,^7,8^ 22.9% in a peri-urban,^9^ and 7.1% in a rural^10^ ART clinic population, respectively.

Chronic HBV may complicate the administration of ART for HIV, because of the three-fold increase in the incidence of antiretroviral (ARV) hepatotoxicity with HIV-HBV co-infection as well as the more complex selection of drugs to avoid resistances.^4,6,11^ Lamivudine, emtricitabine and tenofovir have been shown to possess both anti-HIV and anti-HBV properties.^3^ As lamivudine has a comparatively low genetic barrier to viral resistance, monotherapy with this drug should be avoided.^12^

### Management of hepatitis B virus-HIV co-infection in South Africa

Based on evidence on the efficacy of tenofovir against HBV,^11^ the potential problem of HBV monotherapy with the existing first-line ART regimen and an estimated 5% HIV-HBV co-infection rate, the Provincial Government of the Western Cape province in South Africa introduced a policy on hepatitis B screening of HIV patients and the substitution of tenofovir for stavudine in July 2008.^13^ Subsequently, all patients attending the ART clinics to initiate treatment were to be screened using the serum HBsAg test. This testing policy was terminated in 2010, when new national South African Antiretroviral Treatment Guidelines introduced tenofovir as recommended first-line treatment in place of stavudine. With two HBV-active substances now part of the first-line HIV treatment, hepatitis B screening was no longer recommended during baseline workup. However, the period of HBsAg screening provided a window of opportunity for retrospective ‘data mining’ to establish an estimate for the local positivity rate amongst the HIV-infected population.

An active hepatitis B vaccination has been part of the South African childhood vaccination programme since 1995, giving hope that the rate of co-infection in the younger population will decrease significantly. With regards to screening recommendations, the most recent (2014) HIV treatment guidelines by the Southern African HIV Clinicians Society recommend HBsAg screening for all newly diagnosed HIV patients.^14^ Those with chronic HBV (positive HBsAg) qualify for ART initiation, and ‘eligible’ (negative) adults should be considered for vaccination. The national HIV treatment guidelines by the South African Department of Health (2014) include HBsAg screening and ART initiation of co-infected patients, but do not mention the option of vaccination.^15^

## Methodology

A retrospective, observational and cross-sectional study was performed. Approval was obtained from the Health Research Ethics Committee of Stellenbosch University (N10/11/360) and the Department of Health of the Provincial Government of the Western Cape. The study population consisted of all adult (aged 18 years and older) HIV patients presenting to the TC Newman ART Centre for ART initiation from 01 October 2008 until end of April 2010, when a new ART protocol was introduced and the routine HBsAg screening was omitted. Serum was routinely tested for HBsAg as part of baseline screening tests as per provincial protocol, and the results were available retrospectively. No additional expenditure for laboratory testing was incurred. Information regarding all patients started on ART at the TC Newman ART Centre was available from an existing database. TC Newman is a community health centre with a dedicated ARV Clinic, located in the Cape Winelands town of Paarl, about 40 km from Cape Town. From 2004 to 2010, approximately 2700 patients had been started on ART.

The relevant data (gender, age, CD4 counts and HBsAg) for the study population were retrieved and the anonymised data were entered into a MS Excel spreadsheet and analysed by a statistician using STATISTICA version 8 (StatSoft Inc. 2008). As the HBsAg results were not routinely captured on the database, the results had to be retrieved from the National Health Laboratory Services’ database (WWDisa). For those patients (185) where no results could be found on this system, patients’ folders had to be retrieved and reviewed manually.

Summary statistics were used to describe the variables. Distributions of variables were presented with histograms and frequency tables. Relations between input and response variables were analysed using appropriate statistical tools (analysis of variance and likelihood ratio chi-square tests), as applicable. A *p*-value of < 0.05 was considered to represent statistical significance in hypothesis testing, and 95% confidence intervals were used to describe the estimates of unknown parameters.

## Results

### Demographics of research sample

Out of a total of 569 patients examined for initiation of highly active antiretroviral therapy at TC Newman (i.e. approximately 30 per month), 498 had hepatitis B results available and were included in the study population. Of the 71 patients (12.4%) who had no hepatitis B result available, folders for 7 patients could not be located, whilst 64 had not been tested, most of them during the phasing-in and -out of the protocol in the first and last months. A breakdown of the basic demographics of the study sample is given in Table 1.

**TABLE 1:** Demographics and baseline values of the sample.

Demographics	Percentage of total sample	Age, mean ± SD	CD4 count at baseline	Median and quartiles	HBsAg+ (%)	*n*
Male (*n* = 199)	40	38 ± 9.8	150	70–199	9.0	18
Female (*n* = 299)	60	33 ± 8.8	165	104–206	6.60	20

**Total (*n* = 498)**	**100**	**39 ± 9.0**	**159**	**91–204**	**7.60**	**38**

### Hepatitis B surface antigen positivity rates

**FIGURE 1:**
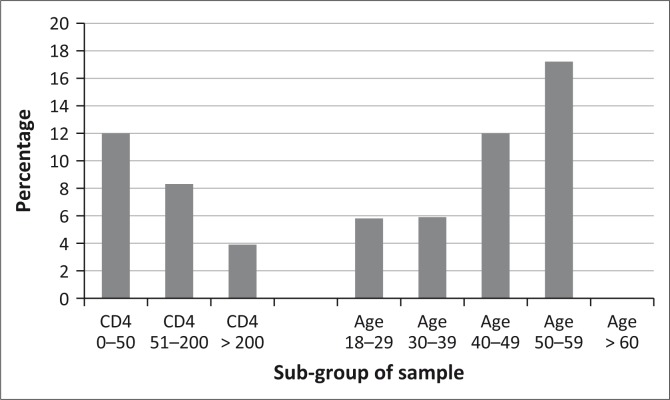
Hepatitis B surface antigen positivity for different CD4 counts and age groups in the study population.

The overall prevalence for HBsAg positivity was 7.6%, with a prevalence amongst male patients of 9% (18/199) and amongst female patients of 6.6% (20/299); however, the difference was not statistically significant (*p* = 0.33). The highest prevalence of hepatitis B was found in the age group 50–59 years of 17.2% (5/29), followed by 12% (12/100) in the age group 40–49, 5.9% (13/220) in the age group 30–39, 5.8% (8/137) in the age group 18–29 and 0% (0/10) in the age group 60–69 (Figure 1). This tendency of correlation with age was not significant (*p* = 0.076).

In the group with the lowest CD4 count, the highest prevalence of 12% was found, with the next highest of 8.3% in those with a CD4 count of 51–200. No significant correlation between HBsAg positivity and CD4 count occurred (*p* = 0.1).

## Discussion

The prevalence of HBsAg positivity in HIV-positive patients of 7.6% is comparable to those rates found in studies in the northern hemisphere as well as to those in a number of South African treatment cohorts, with most of the studies showing rates between 5% and 9%, with only two ‘outliers’ around 20%.^4,5,6,7,8,9,10^ Also in keeping with the generally documented trends are our findings of higher co-infection rates amongst males and in individuals with lower CD4 counts.

The rate of 7.6% of active chronic infections found in this study might reflect a higher rate of chronification of HBV infections after acute infections in the HIV-positive population, as suggested in the literature, but might also be due to increased risk behaviour or exposure that led to the HIV infection.

HIV-HBV co-infections constitute a significant problem in our local population, with 1 in 12 patients who start ART being co-infected. Against the background of the current treatment guidelines,^14,15^ these cases will be picked up if the guidelines are adhered to. As the two first-line HIV drugs tenofovir and lamivudine (or its ‘equivalent’, emtricitabine) are both active against HBV, the clinical relevance of these co-infections arises once the decision to discontinue tenofovir would be on the cards. This might either be the case in HIV treatment failure with the first-line regimen or if tenofovir needs to be discontinued because of its nephrotoxicity, e.g. in renal failure or the concurrent use of other nephrotoxic drugs such as second-line TB drugs. Whilst it is appropriate in the former scenario to continue tenofovir together with the new (second-line) ARV regimen, the latter scenario is more complicated and would need expert advice to balance harm and benefit.

The finding that the majority of our study population had a CD4 count below 200 can be explained by the fact that the major trigger for treatment initiation in the 2008 national ARV protocol was a CD4 count below this threshold.

### Strengths and limitations of our study

The fairly complete cohort is a strength of this study, with only 12% of the study population not having HBsAg results available. Limitations are the selection of the study population. As all patients required ARV treatment, they represent a sample of advanced HIV infection. The (opportunistic) use of existing laboratory data is a further limitation, as the screening with HBsAg is unable to detect so-called ‘occult’ infections and no further serological tests could be performed on the samples.

## Conclusion

In this study, we found the overall prevalence of hepatitis B antigen positivity amongst HIV-positive adult patients starting ART in Paarl to be 7.6%. There was a trend towards a higher prevalence amongst men, the age group 50–59 as well as those with a CD4 count below 50 µL; however these findings were not statistically significant. ‘Opportunistic’ research, using existing data collected as part of routine screening policies, can successfully be used to acquire relevant epidemiological information. A reasonably good compliance with the testing policy was found, with only 11.2% of the study population not having had a HBsAg performed, whilst 1.2% of the folders could not be located.
